# Ageism toward older workers and wellbeing at work: a systematic review

**DOI:** 10.3389/fpsyg.2026.1824641

**Published:** 2026-06-10

**Authors:** Andy Luis Marrero-Vega, Jesus Yeves, Mariana Bargsted, Sebastián Saéz

**Affiliations:** 1 Programa de Doctorado en Psicología, Facultad de Psicología, Universidad Diego Portales, Santiago, Chile; 2 Programa de Estudios Psicosociales del Trabajo (PEPET), Facultad de Psicología, Universidad Diego Portales, Santiago, Chile; 3 Núcleo Milenio sobre la evolución del trabajo (MNEW), Facultad de Psicología, Universidad Diego Portales, Santiago, Chile

**Keywords:** ageism, older workers, systematic review, wellbeing, workplace

## Abstract

**Introduction:**

In the context of unprecedented workforce age diversity, workplace ageism is increasingly reported. This review synthesizes empirical evidence on the relationship between workplace ageism and workplace wellbeing among workers aged 50 and older, focusing on how these effects unfold (mediation) and the conditions under which they are amplified or buffered (moderation).

**Methods:**

A systematic literature review was conducted following PRISMA guidelines. Searches were conducted in major academic databases, including Web of Science, Scopus, Pubmed, among others. Of the 3,378 records identified, 22 met the inclusion criteria.

**Results:**

Across studies, ageism showed a consistently negative and statistically significant association with workplace wellbeing outcomes, even though ageism and wellbeing were measured with heterogeneous indicators. Findings also suggest spillover beyond work, for example, via decreases in life satisfaction or subjective wellbeing. Moderation evidence was limited; when tested, social support (supervisor and coworker support) emerged as the only moderator in this association. Mediation evidence was more developed and clustered around three pathways: (1) cognitive processes (e.g., appraisals and rumination); (2) affective processes (e.g., aging anxiety); and (3) job demands–resources mechanisms.

**Conclusions:**

By clarifying these moderators and mechanisms, the review points to leverage points for practices that foster inclusive, intergenerational workplaces and safeguard wellbeing.

## 1 Introduction

The aging of the global population has produced what is arguably the most age-diverse workforce in modern history (Rudolph and Zacher, 2015), and older adults now represent an increasing share of the labor force worldwide (OECD, 2025). As the workforce age diversity continues to expand, manifestations of ageism in the workplace have become more prevalent (Cebola et al., 2021). Consequently, older workers, defined as those 50 years and over (International Labour Organization [ILO], 2011; OECD, 2006), often face disadvantages in the workplace. These conditions undermine the dignity and rights of older workers and limit their potential to experience wellbeing.

Ageism is a multifaceted construct that has been defined in diverse ways across the literature (Iversen et al., 2009). According to the Pan American Health Organization (PAHO) (2021), it encompasses how individuals think, feel, and behave toward others or themselves based on age. In the workplace, it often undermines the visibility of employees' skills and competencies (Posthuma and Campion, 2009; Posthuma et al., 2012), increases perceived stress, psychological disengagement, and increases intentions to leave the workforce (Lagacé et al., 2023; Purchase et al., 2024; Vickerstaff and Van der Horst, 2021). Furthermore, it also influences organizational processes, such as recruitment, career advancement, and performance evaluations, often placing older employees at a disadvantage despite their qualifications (Bal et al., 2011; Cebola et al., 2021; Petery and Grosch, 2022). These dynamics compromise wellbeing at work (Kang and Kim, 2022), understood as the combination of positive and negative emotional experiences at work, alongside the cognitive evaluation of job satisfaction (Page and Vella-Brodrick, 2008).

Although some previous reviews have explored the association between age discrimination and wellbeing across various contexts (e.g., Kang and Kim, 2022), they often overlook the specificity of the work environment. Prior research has also focused on work-related ageism (Bae and Choi, 2022; Cebola et al., 2021) or on ageism in personnel management practices, such as hiring (Batinovic et al., 2023). However, less attention has been given to the specific relationship between ageism directed at older workers and workplace wellbeing, despite its growing relevance in an aging labor force (Cebola et al., 2021; Kang and Kim, 2022). There is also a need to expand knowledge regarding the moderators and mediators that explain how this relationship unfolds and identify leverage points for promoting wellbeing.

This review aims to identify and synthesize the available empirical evidence concerning the relationship between ageism directed at older workers and their workplace wellbeing. To this end, a comprehensive and methodologically rigorous literature search was conducted to address the following two questions: (1) What is the relationship between ageism and workplace wellbeing among employees aged 50 years and older? And (2) Which variables function as mediators and moderators in the relationship between ageism and workplace wellbeing in these employees?

## 2 Theoretical background

### 2.1 A brief exploration of the concept of ageism

Ageism encompasses stereotypes, prejudices, and discriminatory practices directed against others or self-inflicted based on age (Iversen et al., 2009; Pan American Health Organization [PAHO], 2021), expressed in both conscious and unconscious ways. It manifests through stereotypes, which are mental frameworks that influence beliefs and expectations about specific age groups; and prejudices, which are mostly emotional reactions toward individuals of a certain age; discrimination, on the other hand, refers to actions that either disadvantage or advantage people based on age (Iversen et al., 2009; Pan American Health Organization [PAHO], 2021).

Also, ageism manifests at three distinct levels of expression: intrasubjective, intersubjective, and institutional. At the intrasubjective level, individuals integrate negative social representations of aging into their self-concept (Pan American Health Organization [PAHO], 2021). Across the life course, exposure to stereotypes about aging influences the internalization of specific attitudes toward age through a cognitive and motivational process that seeks to bring order to the concept of self and individual behavior (Levy, 2001). Once the individual internalizes this identity, it acts as a set of rules that dictates what “a person like me” (e.g., an older worker or aging person) should or should not do in a given situation (Ashforth and Mael, 1989; Hogg and Terry, 2000). This often results in self-directed ageist attitudes that negatively affect work performance and personal health (Levy, 2001; Levy et al., 2002, 2011).

The intersubjective level arises from interactions among two or more individuals (Pan American Health Organization [PAHO], 2021). This dimension encompasses both intragenerational and intergenerational exchanges (Ayalon and Tesch-Römer, 2018; Butler, 1969, 2008) and enables us to distinguish between those who perpetuate ageism and those who experience its effects. In this context, in-group and out-group boundaries are primarily defined by generational belonging, implying that age becomes a significant category through which differentiation is constructed at work (Ayalon and Tesch-Römer, 2018). This dynamic of comparison and differentiation accentuates generational differences (Ayalon and Tesch-Römer, 2018; Tajfel and Turner, 1979). In the workplace, this process enables employees to construct their subjectivity and influences their choice of activities that align with the salient aspects of their identity (e.g., age-based exclusion processes).

Building on these interpersonal interactions, the institutional level encompasses the broader context of laws, rules, regulations, policies, and organizational practices that unjustly limit opportunities and systematically disadvantage individuals based on age (Pan American Health Organization [PAHO], 2021). This level serves as the organizational framework within which workplace elements, such as culture, practices, and work design, function as either resources that enhance wellbeing or demands that diminish it (Bakker and Demerouti, 2013; Demerouti et al., 2001).

Although this multidimensional and multilevel understanding of ageism has gained increasing conceptual acceptance (Cadiz et al., 2024; Finkelstein et al., 2018; Previtali et al., 2022), the way ageism is operationalized in empirical research remains heterogeneous (Ayalon et al., 2019; Iversen et al., 2009; Yeves et al., 2026). As a result, studies conducted under the same label may capture different dimensions or levels of the phenomenon, complicating comparisons across findings and the identification of more precise explanatory mechanisms.

### 2.2 What implies wellbeing at work?

Wellbeing at work remains theoretically broad and operationally dispersed, with limited consensus regarding its exact definitions, boundaries, and measurement (Taris and Schaufeli, 2015; Bautista et al., 2023). In this systematic review, workplace wellbeing is understood as a multidimensional, situated construct expressed within the specific domain of work and organized as a higher-order factor comprising positive and negative affect at work, and job satisfaction (Cotton and Hart, 2003; Page and Vella-Brodrick, 2008).

It is therefore distinct from general wellbeing, given the unique experiences and complexities associated with work life (Daniels, 2000; Warr, 1990; Zheng et al., 2015). This distinction deserves to be made more explicit because workplace wellbeing overlaps with, but is not equivalent to, broader constructs such as subjective and psychological wellbeing. Subjective wellbeing usually refers to individuals' overall evaluations of their lives and their affective balance across life domains (Diener, 1984; Diener et al., 1999), whereas psychological wellbeing tends to emphasize broader functioning, self-acceptance, purpose, and personal growth (Ryff, 1989; Ryff and Keyes, 1995). By contrast, workplace wellbeing is anchored specifically in the occupational domain. In that sense, it captures how individuals feel and evaluate their work experience, rather than their life circumstances more generally. This distinction is especially relevant in the present review, given that part of the literature on ageism has relied on broader wellbeing indicators that are conceptually close, but not strictly equivalent, to wellbeing at work.

Regarding cognitive evaluations, job satisfaction plays an important role. It refers to an individual's personal evaluation of their work and their level of satisfaction with their work environment (Cotton and Hart, 2003; Spector, 1997). Historically, job satisfaction became the dominant indicator of wellbeing in organizational research (Staw, 1984). From a transactional perspective, these judgments can be understood as cognitive appraisals of the person–environment relationship at work, in which employees evaluate the significance of working conditions for their goals and wellbeing (Lazarus and Folkman, 1984).

However, the approach of using this variable as the unique measure of wellbeing at work has been questioned (Wright and Cropanzano, 2000, 2004), among other reasons, because it ignores the affective component of this experience (Daniels, 2000; Warr, 1990). Regarding the emotional aspect of workplace wellbeing, workplace events actively trigger affective states that, over time, can influence employees' attitudes and behaviors, either positively or negatively (Weiss and Cropanzano, 1996). In this sense, the literature has distinguished between positive and negative affect related to work (Daniels, 2000; Page and Vella-Brodrick, 2008; Warr, 1994, 1999). Positive affect at work refers to a pleasant emotional state characterized by feelings such as joy, satisfaction, enthusiasm, and vitality that are specifically associated with the work experience. Negative affect at work denotes an unpleasant emotional state that includes anxiety, anger, frustration, despondency, or sadness in response to work-related situations. Furthermore, it is essential to recognize that older workers tend to exhibit distinct emotional resources for coping with challenging workplace situations due to their accumulated life experiences (Charles, 2010).

Although wellbeing at work is a desirable goal, achieving it is often hindered by relational and structural factors that operate as obstacles within the labor market. Among these, age has emerged as a central axis of differentiation and stratification at work (Krekula et al., 2018). In this context, one category that helps us make sense of this sociopsychological dynamic and its implications is ageism. Studying ageism matters because it has strong explanatory potential for anticipating worse psychosocial outcomes at work, including decreases in wellbeing, particularly in the context of an aging workforce. Accordingly, this paper argues that ageism, expressed at its various levels (Iversen et al., 2009; Pan American Health Organization [PAHO], 2021), constitutes a fundamental barrier to achieving wellbeing at work, especially for older workers.

## 3 Methods

### 3.1 Search strategy

A systematic literature search was conducted in accordance with PRISMA guidelines (Moher et al., 2009). The search was performed in Web of Science, Scopus, SciELO, PubMed, PsycInfo (EBSCO), Psychology and Behavioral Sciences Collection (EBSCO), and Psychology Database (ProQuest). Search terms were developed based on the PICOS framework. They were organized into four blocks: population (older workers), exposure (ageism), outcome (wellbeing), and setting (work-related context). Synonyms within each block were combined using “OR”. The search blocks were combined using the “AND” operator. The syntax was adapted to each database's indexing system and interface. Table 1 presents the PICOS-derived search term blocks used to construct the database-specific strategies.

**Table 1:** PICOS framework for systematic review and search terms.

Attribute	Characteristics	Search terms
Population of interest	Older workers over 50 years old	“older worker” OR “older adults” OR “older people” OR aged OR “age-related” OR ageing OR aging OR senior OR elder.
Intervention (exposure)	Exposure to ageism	ageism OR agism OR agist OR ageist OR “age discrimination” OR “age stereotype” OR “age prejudice” OR “ageist attitude” OR “self perceptions of aging” OR “self perceptions of ageing” OR “age bias” OR stereotype OR prejudice OR discrimination OR stigma.
Comparator	Not apply	Not apply.
Outcome of interest	Wellbeing	wellbeing OR wellbeing OR “well being” OR “laboral wellbeing” OR “labour wellbeing” OR “work wellbeing” OR “employee wellbeing” OR “workplace wellbeing” OR “work-related wellbeing” OR “occupational wellbeing” OR “life wellbeing” OR “organizational wellbeing” OR “psychological wellbeing” OR “subjective wellbeing” OR “experiential wellbeing” OR “work engagement” OR “job satisfaction” OR “employee satisfaction” OR “work satisfaction” OR “career satisfaction” OR “quality of life.”
Settings	Workplace	“late career” OR late-career OR “career stage” OR career OR workers OR workplace OR workforce OR “labour market” OR “labor-market” OR “work sector” OR “work field” OR “job sector” OR “job type” OR employment OR employee OR staff OR personnel.

Only studies published from 1970 onwards were considered, as Butler's (1969) seminal publication introduced the term “ageism.” The final search update was conducted on September 30, 2024. All records were exported to The EndNote Team (2013) for reference management and duplicate handling and subsequently imported into Covidence systematic review software (2024) for screening and study selection. No separate systematic search of gray literature was conducted. Nonetheless, one source (Landrum, 2000), classified as gray literature, was identified through the main search and study selection process and included in the final sample. This review was not prospectively registered in PROSPERO or in any other systematic review registry.

### 3.2 . Study selection

Table 2 details the inclusion and exclusion criteria for the identified records, based on the PICOS framework. Among the additional inclusion criteria, only empirical studies were considered eligible for review. Nonempirical studies (such as theoretical discussions, position papers, government reports, protocols, opinion pieces, or book reviews), literature reviews, scoping reviews, systematic reviews, or meta-analyses were not considered. Studies with broader age ranges were considered eligible only when they included workers aged 50 and older and reported findings disaggregated by that age group, either through subgroup analyses, age-based comparisons, or results directly interpretable for workers in later career stages. Additionally, we applied other exclusion criteria, including studies not published in English or Spanish, studies with inaccessible full texts, and studies published before 1970.

**Table 2:** PICOS criteria for inclusion and exclusion.

Attribute	Characteristics	Inclusion criteria	Exclusion criteria
Population of interest	Older workers over 50 years old.	Individuals aged 50 years or older who are employed.	Samples consisting of older adults who are unemployed, economically inactive, retired, and relying solely on a pension, or engaged exclusively in volunteer work.
Intervention (exposure)	Exposure to ageism	Studies focusing on ageism towards older workers in the late stage of their careers.	No measure or operationalization of ageism.
Comparator	Not apply	Not apply	Not apply
Outcome of interest	Wellbeing	Studies examining the relationship between ageism and workplace wellbeing.	Outcomes unrelated to wellbeing
Settings	Workplace	Studies conducted in workplace environments.	Contexts in which persons over 50 are service users or patients, not employees.

### 3.3 Selection process

**Figure 1:**
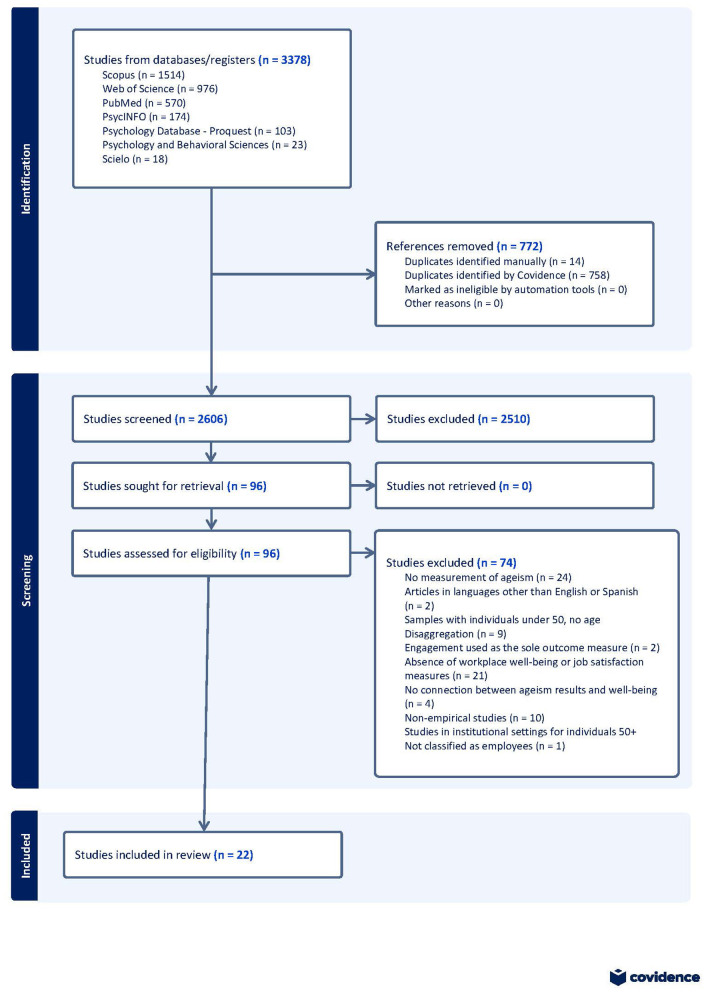
Search and screening flow diagram. Flow diagram provided by Covidence systematic review software (2024).

After eliminating duplicates identified by Covidence systematic review software (2024) (*n* = 758), four researchers independently conducted an anonymous review of 2,606 titles and abstracts using the platform. Each article received two independent reviews. Any conflicts that arose during the review process were resolved through consensus with the assistance of a third reviewer. During the initial screening phase, most studies were excluded for not meeting the inclusion criteria (*n* = 2,510). Additionally, 14 studies were manually identified as duplicates. This process resulted in the selection of 96 studies for further full-text eligibility assessment. The same four reviewers subsequently analyzed the full texts of the selected studies, ensuring that each article received two independent, blinded evaluations. Discrepancies were resolved through a third reviewer's revision, resulting in the inclusion of 22 articles that met the inclusion criteria, while 74 articles were excluded for not meeting the criteria. The PRISMA flowchart shown in Figure 1 illustrates the process described earlier.

### 3.4 Data collection process

After review of the full text, the following data were extracted from each of the 22 studies: (1) general information (country in which the study was conducted, language of the article, study setting, type of publication, and author's contact details); (2) methods (study aim, study design, data collection methods and sampling methods); (3) population characteristics (e.g., total sample size, mean age, age range, gender, and education level); (4) measures and results of the exposure phenomena—ageism—(classification, measure, and results); (5) measures and results of the exposure phenomena—workplace wellbeing—(classification, measure and results). In addition, we identified the (6) results of the relationship between exposure and outcome, including any mediators or moderators, if applicable. We also assessed the nature of the established relationship (e.g., correlation, moderation, mediation, or causality). Furthermore, we considered the following elements: (7) the main findings related to ageism and workplace wellbeing; and (8) the study's limitations and potential avenues for future research.

### 3.5 Study risk of bias assessment

To assess the methodological quality of the included studies, the Joanna Briggs Institute (JBI) critical appraisal checklist for analytical cross-sectional studies was used (Moola et al., 2020). Based on the characteristics of the included studies, this tool was considered the most appropriate. Because the review focused on nonclinical psychosocial phenomena rather than diagnosed health conditions, item 4 of the checklist (“Were objective, standard criteria used for measurement of the condition?”) was considered not applicable across all studies. Therefore, methodological quality was summarized based on the seven applicable criteria.

## 4 Results

### 4.1 Study selection and characteristics

This systematic review synthesizes findings from 22 studies to address the research question regarding the relationship between ageism and workplace wellbeing for employees aged 50 and older. The articles included in this review were published between 2000 and 2024 and analyzed diverse samples from various countries.

All studies were classified as peer-reviewed journal articles, except for one case, a doctoral thesis by Landrum (2000), classified as gray literature. Notably, all these, including the thesis, were originally written in English. The most frequently cited countries were the United States (8), Australia (3), Japan (2), Spain (2), Italy (2), Denmark (2), and Slovenia (2). Other countries, including China, Korea, Saudi Arabia, Israel, and several additional European Union member states, were represented in only one study each. Notably, no Latin American countries were represented in this review, despite using the SciELO database and considering Spanish a relevant language in the inclusion criteria.

The sample sizes demonstrate a high degree of heterogeneity, ranging from 20 participants in a qualitative study (Mohamed and Shaban, 2024) to 32,984 participants in a quantitative prevalence study (Lee et al., 2017). Most studies involve sample sizes ranging from 100 to 3000 participants. The age range in studies also varied significantly, from 15 (Lee et al., 2017) to 95 years (Suberry and Bodner, 2024). Despite this dispersion in participants' ages, this review focused only on older workers aged 50 and above. Regarding gender distribution, most samples showed a higher proportion of women, indicating a slight, and in some cases marked, overrepresentation of female participants (e.g., Carral and Alcover, 2019; Macdonald and Levy, 2016). Notable exceptions include one study that relied on a sample of men aged 55–64 (Harada et al., 2019) and one study that used exclusively female samples (Shippee et al., 2019).

In addition to these general characteristics, the included studies also varied substantially in study design, age definitions, and the operationalization of both ageism and wellbeing outcomes. Table 3 summarizes these sources of methodological heterogeneity and their implications.

**Table 3:** Methodological heterogeneity across included studies.

Dimension	Heterogeneity observed	Examples	Implication
Study design	The evidence base was dominated by cross-sectional and single-wave correlational studies, with fewer longitudinal studies and one qualitative study.	Cross-sectional studies: e.g., Alam and Shin (2020), Carral and Alcover (2019), Harada et al. (2019). Longitudinal studies: e.g., Griffin et al. (2016), Marchiondo et al. (2019), Shippee et al. (2019), von Hippel et al. (2019). Qualitative study: e.g., Mohamed and Shaban (2024).	Limits causal inference and temporal interpretation, and makes it difficult to distinguish directionality from association.
Age definition/age inclusion criteria	Definitions of “older worker” varied across studies. Some focused explicitly on workers aged 50+, others used 55+ or 60+, and several relied on broader age ranges with subgroup comparisons or age-based analyses.	Workers aged 50+: e.g., Choi et al. (2018), Marrero-Vega et al. (2025). Workers aged 55+ or 60+: e.g., Harada et al. (2019), Landrum (2000), Takeuhi and Katagiri (2024). Broader age ranges with subgroup comparisons: e.g., Lee et al. (2017), von Hippel et al. (2019), Alam and Shin (2020).	Reduces comparability across studies and complicates conclusions about “older workers” as a clearly defined population.
Operationalization of ageism	Ageism was measured predominantly as perceived or experienced age discrimination, although some studies examined stereotype threat, aging anxiety, negative self-perceptions of aging, aging-as-loss, or broader ageism-related experiences.	(Perceived) Age discrimination: e.g., Griffin et al. (2016), Yeung et al. (2021), Roscigno et al. (2022), Thorsen et al. (2016). Stereotype threat: e.g., von Hippel et al. (2013, 2019), Manzi et al. (2019). Aging anxiety/negative self-perceptions: e.g., Macdonald and Levy (2016), Takeuhi and Katagiri (2024), Suberry and Bodner (2024).	Indicates that not all studies assessed the same dimension of ageism, which limits direct comparability and weakens construct equivalence.
Operationalization of wellbeing outcomes	Outcomes ranged from work-specific indicators (e.g., job satisfaction and work-related affect) to broader psychological wellbeing, life satisfaction, depressive symptoms, and adjacent work-related outcomes.	Job satisfaction: e.g., Harada et al. (2019), Macdonald and Levy (2016). Work-related affects: e.g., Takeuhi and Katagiri (2024), Spoelma and Marchiondo (2024). Psychological wellbeing: e.g., Suberry and Bodner (2024), Zhang and Gibney (2019). Life satisfaction: e.g., Shippee et al. (2019). Depression/anxiety: e.g., Landrum (2000). Work strain/engagement: e.g., Yeung et al. (2021).	Blurs the distinction between workplace wellbeing and broader psychological or mental health outcomes, limiting conceptual comparability across studies.
Measurement strategy	Most studies relied on self-report measures; some used brief or single-item indicators, whereas others used validated multi-item scales.	Self-report measures: most studies.Single-item indicator: e.g., Lee et al. (2017), Macdonald and Levy (2016), Roscigno et al. (2022). Validated scales: e.g., Carral and Alcover (2019), Suberry and Bodner (2024).	Introduces variability in measurement precision and construct coverage and increases the risk of common-method bias.

### 4.2 Methodological quality

Methodological quality was assessed using the JBI checklist (Moola et al., 2020). Table 4 summarizes the appraisal of the 22 included studies. Because item 4 was considered not applicable across all studies, quality scores are reported out of seven applicable criteria. Scores ranged from 4 to 7, with a mean of 6.45. Most studies (18 of 22; 81.8%) met 6 or 7 of the 7 applicable criteria, indicating generally adequate methodological quality across the evidence base.

**Table 4:** Review of methodological quality.

Reference	1	2	3	4	5	6	7	8
Landrum (2000)	Y	Y	U	N/A	Y	Y	Y	Y
Takeuhi and Katagiri (2024)	Y	Y	Y	N/A	Y	Y	Y	Y
Suberry and Bodner (2024)	Y	Y	Y	N/A	Y	Y	Y	Y
Alam and Shin (2020)	N	Y	Y	N/A	Y	Y	Y	Y
Mohamed and Shaban (2024)	Y	Y	Y	N/A	N	N	Y	Y
Roscigno et al. (2022)	Y	Y	Y	N/A	Y	Y	Y	Y
Carral and Alcover (2019)	Y	Y	Y	N/A	Y	Y	Y	Y
Choi et al. (2018)	Y	Y	Y	N/A	U	U	Y	N
Griffin et al. (2016)	Y	Y	Y	N/A	Y	Y	Y	Y
Harada et al. (2019)	Y	Y	Y	N/A	Y	Y	Y	Y
Jelenko (2020)	Y	Y	Y	N/A	Y	Y	Y	Y
Lee et al. (2017)	Y	Y	Y	N/A	U	N	Y	Y
Macdonald and Levy (2016)	Y	Y	Y	N/A	Y	Y	Y	Y
Manzi et al. (2019)	Y	Y	Y	N/A	Y	Y	Y	Y
Shippee et al. (2019)	Y	Y	N	N/A	Y	Y	N	Y
Spoelma and Marchiondo (2024)	Y	Y	Y	N/A	Y	Y	Y	Y
Thorsen et al. (2016)	Y	Y	Y	N/A	Y	Y	Y	Y
von Hippel et al. (2013)	Y	Y	Y	N/A	Y	Y	Y	Y
von Hippel et al. (2019)	Y	Y	U	N/A	Y	Y	Y	Y
Yeung et al. (2021)	Y	Y	Y	N/A	Y	Y	Y	Y
Zhang and Gibney (2019)	Y	Y	U	N/A	Y	Y	Y	Y
Marchiondo et al. (2019)	Y	Y	Y	N/A	Y	Y	Y	Y

1: Were the criteria for inclusion in the sample clearly defined?

2: Were the study subjects and the setting described in detail?

3: Was the exposure measured in a valid and reliable way?

4: Were objective, standard criteria used for measuring the condition?

5: Were confounding factors identified?

6: Were strategies to address confounding factors stated?

7: Were the outcomes measured in a valid and reliable way?

8: Was appropriate statistical analysis used?

Y, Yes; N, No; U, Unclear; N/A, Not applicable.

All studies met criterion 2 (“Were the study subjects and the setting described in detail?”), indicating adequate reporting of study context and participants. Most studies also met criterion 8, indicating appropriate statistical analysis (95.5%), and criterion 7, indicating valid and reliable outcome measurement (95.5%). Exposure measurement (criterion 3) was rated as valid and reliable in 81.8% of studies. Confounding was handled less consistently: confounders were identified in 86.4% of studies (criterion 5), and strategies to address them were reported in 86.4% (criterion 6). Instances of “No” or “Unclear” ratings were concentrated mainly in exposure measurement and confounding-related criteria, with fewer limitations observed for outcome measurement and statistical analysis.

### 4.3 Relationship between ageism and workplace wellbeing

Across the revised studies, ageism was defined and measured mostly as perceived age discrimination at work (or only age discrimination), in 18 studies of a total of 22 (e.g., Carral and Alcover, 2019; Choi et al., 2018; Macdonald and Levy, 2016; Marchiondo et al., 2019). A second group of studies captured ageism through stereotypical processes, mainly via age-related stereotype threat framed as an event or appraisal (Manzi et al., 2019; Spoelma and Marchiondo, 2024; von Hippel et al., 2013, 2019) and, to a lesser extent, through indicators of negative self-perceptions of age (Takeuhi and Katagiri, 2024) and aging understood as loss (Suberry and Bodner, 2024). At the affective dimension of prejudice, ageism was measured as aging anxiety, understood as anxiety about one's own aging (Macdonald and Levy, 2016; Takeuhi and Katagiri, 2024). Finally, only one qualitative study used a broader category of “experiences of ageism” without disaggregating it into specific dimensions (Mohamed and Shaban, 2024), a label that does not appear in the other studies in the review and may reflect the qualitative nature of this particular study.

The reviewed studies show that workplace ageism is a prevalent experience among older workers and is consistently documented across diverse organizational contexts. Beyond its prevalence, the evidence suggests a systematic relationship between ageism and multiple indicators of workplace wellbeing. Most commonly, this association is documented when workplace wellbeing is assessed through its core dimensions. In this sense, ageism is systematically related to lower job satisfaction, an association found in 11 of the reviewed studies (e.g., Griffin et al., 2016; Harada et al., 2019; Macdonald and Levy, 2016; von Hippel et al., 2013). Related to the affective dimension, which has been less studied, ageism is associated with less positive and more negative affects at work (Mohamed and Shaban, 2024; Takeuhi and Katagiri, 2024), as well as with a depletion of emotional resources at work (Spoelma and Marchiondo, 2024; Yeung et al., 2021).

This association also holds when wellbeing is measured in global, nondomain-specific terms. Across the reviewed studies, workplace ageism is consistently linked to lower overall wellbeing, even when the outcome is assessed through general indicators not specifically tied to the work context (Lee et al., 2017; von Hippel et al., 2019). Also, a group of studies conceptualizes wellbeing more as an adaptive capacity, showing negative associations between ageism and psychological wellbeing (Suberry and Bodner, 2024; Zhang and Gibney, 2019). In addition, broader evaluative outcomes point in the same direction: ageism is associated with lower life satisfaction and subjective wellbeing (Carral and Alcover, 2019; Shippee et al., 2019; Takeuhi and Katagiri, 2024).

A wider range of outcomes further reflects this adverse association between ageism and wellbeing at work. These include general positive psychological states such as flourishing (Manzi et al., 2019) and work enjoyment (Choi et al., 2018), mental health outcomes such as depression (Landrum, 2000; Marchiondo et al., 2019; Shippee et al., 2019) and anxiety (Landrum, 2000), as well as physiological stress responses such as somatic complaints (Spoelma and Marchiondo, 2024). This pattern also extends to other work-related outcomes closely connected to wellbeing, including work engagement, intention to stay, and work strain (Yeung et al., 2021), as well as job engagement (Macdonald and Levy, 2016), job-related stress (Roscigno et al., 2022), and even early retirement (Thorsen et al., 2016; Yeung et al., 2021), among other outcomes.

### 4.4 Mediators in the association between ageism and workplace wellbeing

Taken together, the reviewed studies suggest that mediation evidence clustered around two pathways: (1) affective and cognitive processes and (2) job demands and resources mechanisms. Within the affective processes, the associations between workplace ageism and life satisfaction, and between workplace ageism and negative affect, were partially mediated by aging anxiety (Takeuhi and Katagiri, 2024). Among the cognitive processes, this same association was also partially mediated by negative self-perception of age (Takeuhi and Katagiri, 2024). Also, the association between stereotype threat event and wellbeing was partially mediated by challenge appraisals and rumination (von Hippel et al., 2019). In addition, the relation between workplace age discrimination and somatic complaints and emotional exhaustion was partially mediated by challenge and threat appraisals (Spoelma and Marchiondo, 2024).

Mediation evidence was also consistent with job demands and resources mechanisms. In one study, perceived financial strain fully mediated the association between perceived age discrimination and depressive symptoms, while it partially mediated the association between perceived age discrimination and life satisfaction (Shippee et al., 2019). In addition, job resources and demand-related mechanisms explained part of the association between perceived age discrimination and work outcomes; the relationship with work engagement was partially mediated by job resources (supervisor and coworker support) and by demand-related mechanisms (workload and emotional exhaustion). In contrast, the association with intention to stay was partially mediated by job resources (supervisor and coworker support) (Yeung et al., 2021). Finally, the association between perceived age discrimination and work strain was partially mediated by demand-related mechanisms, with the indirect effect emerging specifically through emotional exhaustion (Yeung et al., 2021).

### 4.5 Moderators in the association between ageism and workplace wellbeing

Overall, the evidence of moderation across the reviewed studies was scarce, converging on relational workplace resources. Harada et al. (2019) examined workplace social support (from supervisors and coworkers) as a moderator of the association between perceived age discrimination and job satisfaction, and their results supported a buffering pattern; the negative association between age discrimination and job satisfaction was weaker when social support was higher. Aligning with this, Roscigno et al. (2022) found that poor supervisory relations act as a moderator of the links between experienced age discrimination and both work-related stress and mental health. Their results indicate an amplifying pattern; when supervisory relations were poorer, the association between age discrimination and higher stress and worse mental health became stronger. Taken together, these findings indicate that, within the available evidence, moderation is most consistently explored through the quality of social support and supervisory relations, highlighting that relational resources in the workplace may condition the implications of ageism for workplace wellbeing.

## 5 Discussion

This systematic review highlights ageism as a relevant psychosocial factor consistently related to poorer workplace wellbeing among employees aged 50 years and older. This pattern emerges despite the conceptual and operational heterogeneity that characterizes the assessment of workplace wellbeing across studies. Similar observations have been reported in recent reviews (Bautista et al., 2023; Iasiello et al., 2024), and the studies included in the present review likewise point to a consistent negative association. This convergence matters because it suggests that the link between ageism and diminished workplace wellbeing is unlikely to be an artifact of any single definition or instrument.

At the same time, the breadth of outcome measures used across studies deserves attention. Some studies assessed workplace wellbeing in a stricter sense, focusing on work-related affect or job satisfaction. Others relied on broader indicators, such as subjective wellbeing, psychological wellbeing, life satisfaction, or mental health symptoms. This heterogeneity suggests that the implications of workplace ageism may extend beyond narrowly defined occupational outcomes. At the same time, it indicates that the evidence is more consistent in supporting a general negative pattern of association than in specifying which dimensions of wellbeing are most directly affected.

This consistency should not be overinterpreted causally. Because the evidence base is composed predominantly of cross-sectional and correlational studies, the review is better positioned to identify recurrent associations than to establish temporal ordering or causal effects. In other words, the current literature supports the conclusion that workplace ageism and poorer wellbeing tend to co-occur. However, it remains less conclusive regarding whether ageism precedes and produces those outcomes, whether lower wellbeing heightens the perception or salience of ageism, or whether both are shaped by unmeasured organizational or individual factors.

Also, it is significant that the evidence is concentrated on intrasubjective and intersubjective expressions of ageism, most often captured as perceived/experienced age discrimination, and, to a lesser extent, as stereotype-based and self-directed processes (e.g., stereotype threat, negative self-perceptions of age, and aging anxiety). In contrast, institutional and organizational manifestations of ageism, such as organizational policies, HR practices, and structural arrangements, remain comparatively underexamined in the reviewed literature.

At an intrapersonal level, the reviewed evidence suggests that workplace ageism is not only an external experience but also a phenomenon that shapes how older workers think, feel, and evaluate themselves at work. This matters because these internalized ageist attitudes are consistently linked to poorer outcomes in domains central to workplace wellbeing. This impact can be understood not only as age discrimination actions, but also in terms of stereotypes and stereotype threat, for example, the fear of confirming negative stereotypes, which shape perceptions and job evaluations and lead to worse workplace wellbeing-related outcomes (Jelenko, 2020; Manzi et al., 2019; von Hippel et al., 2013, 2019). Furthermore, ageism entails a high emotional cost. Prolonged exposure to these negative experiences provokes negative emotions and gradually depletes individuals' coping resources, leading to emotional exhaustion (Spoelma and Marchiondo, 2024). These patterns align with the idea that repeated exposure to ageist events can erode coping resources over time, consistent with the Strength and Vulnerability Integration model (SAVI) (Charles, 2010).

At the intersubjective level, the reviewed evidence reveals a consistent pattern in which ageism manifests in everyday interpersonal and group dynamics at work. This is coherent with the demographic shift described earlier; we now live in the most age-diverse labor force in modern history (Rudolph and Zacher, 2015), and the growing presence of older workers has been identified as a significant determinant of other-oriented ageism in previous research (Marques et al., 2020). Within this context, older people can be more easily framed as a “burden” and as competitors for pensions, employment, and other scarce resources, making negative attitudes toward older adults more salient.

This dynamic is consistent with Social Identity Theory (Tajfel and Turner, 1979), under conditions of heightened intergenerational tension and scarcity framing, behaviors such as microaggressions, marginalization, and exclusion can become social signals that mark “who belongs” and “who does not.” Importantly, our review suggests that these intersubjective dynamics are not just “social background”; they operate as events that function like additional job demands, elevating psychological costs and eroding work resources (Choi et al., 2018; Yeung et al., 2021). At the same time, when positive intergenerational exchanges are supported, especially via supervisor and coworker support, these relational processes can operate more like job resources, interrupting or neutralizing exclusionary signals rather than reinforcing them (Yeung et al., 2021).

Building on these interpersonal interactions, the institutional level can be understood as the “rules of the game” that shape when ageism emerges, how often it recurs, and how strongly it affects workplace wellbeing outcomes. Although the reviewed studies focus mainly on intrasubjective and intersubjective expressions (e.g., perceived/experienced discrimination and stereotype-based processes), our synthesis suggests that organizational arrangements likely operate through at least two linked pathways.

First, organizational policies, enforcement mechanisms, and human resources practices can structure exposure to ageism by legitimizing (or constraining) everyday microexclusions and discriminatory decisions (e.g., in selection, task allocation, development opportunities, and performance management). Second, these same arrangements can shape the conversion of ageism into diminished wellbeing by organizing the distribution of job demands and resources, which are among the mechanisms that appear to mediate and moderate in the reviewed evidence.

For instance, when supervisory relations are poor and accountability is weak, age discrimination may become a recurring job demand that erodes resources and amplifies stress-related outcomes; conversely, when supervisor and coworker support are institutionally enabled (through leadership expectations, workload design, and responsive reporting systems), relational resources can buffer the relation between age discrimination and wellbeing (Harada et al., 2019; Roscigno et al., 2022; Yeung et al., 2021). From this perspective, the expression of ageism at the organizational level is a structural driver that may explain why the ageism–workplace wellbeing association remains consistently negative across heterogeneous outcome definitions and measures.

The mediation evidence in this review clarifies how ageism “gets under the skin” and leads to poorer workplace wellbeing. It does so through two main pathways with clear implications for research and intervention. First, the intrapersonal pathway suggests that workplace ageism is not only something older workers encounter, but something that can be cognitively and affectively processed in ways that shape self-evaluation and emotional functioning over time.

Second, the job demands–resources pathway shows that ageism is both an individual and a job-related phenomenon. Taken together, these two main mechanisms reinforce a core idea, reducing the impact of workplace ageism requires acting both on what ageist experiences trigger within people (self-perceptions, anxiety, appraisals, rumination, etc.) and on the work conditions that allows those experiences (supportive supervision, coworker solidarity, manageable demands, and organizational arrangements that reduce economic vulnerability), because the intrapersonal via and these job-related demand and resources mechanisms appear to operate in conjunction rather than in isolation.

On the other hand, the moderation evidence in this review points to a conceptually important finding: ageism at work does not appear to operate as a uniformly harmful exposure. Its effects do not seem to unfold identically across contexts. Rather, the limited evidence available suggests that its implications for workplace wellbeing depend on the relational conditions of the work environment. Notably, the moderators tested in the reviewed literature are not individual dispositions, but contextual and relational resources, specifically social support from supervisors and coworkers and the quality of supervisory relations (Harada et al., 2019; Roscigno et al., 2022).

Despite the small number of studies, the pattern is theoretically coherent. Supportive relational climates appear to buffer the negative implications of age discrimination, whereas strained or poor-quality supervisory relations appear to amplify them. This suggests that workplace ageism becomes particularly damaging in settings where everyday ties are weak, trust is limited, and supervisory relationships fail to provide protection or recognition. In this way, these findings can also be viewed in the context of broader research indicating that support from supervisors and coworkers serves as a source of meaning at work, as positive interactions may strengthen workers' sense of belonging and inclusion and promote psychological safety, even in the face of age-related threats (Oliveira and Cabral Cardoso, 2018). Viewed from this angle, relational support may not only reduce the impact of ageism as a stressor but also sustain the social and symbolic conditions that enable older workers to continue to experience themselves as legitimate and valued members of the workplace.

At the same time, the moderation literature remains underdeveloped (Batinovic et al., 2023; Petery and Grosch, 2022). The current evidence tells us relatively little about whether the impact of ageism varies across other plausible boundary conditions, such as age subgroup within older workers, occupational position, job insecurity, organizational diversity climate, or the broader institutional context. Thus, the available studies support the view that relational resources matter. However, they do not yet provide a sufficiently broad map of when, for whom, and under what conditions workplace ageism is most harmful. This reinforces the idea that addressing workplace ageism cannot be reduced to changing individual attitudes alone. It also requires strengthening the relational infrastructure of work, including supportive supervision, coworker solidarity, and age-inclusive organizational climates.

### 5.1 . Limitations of the evidence included in the review

The findings of this systematic review should be interpreted in light of the limitations identified in the included studies. Although the methodological quality of the evidence base was generally adequate, it was not free from risk of bias. Most studies met 6–7 of the 7 applicable JBI criteria (18 out of 22), which supports cautious confidence in the overall consistency of the findings. At the same time, incomplete handling of confounding and occasional lack of clarity in measuring exposures and outcomes warrant caution when interpreting the strength and precision of the observed associations. Accordingly, confidence is stronger for the direction and consistency of the association between ageism and workplace wellbeing than for its precise magnitude or causal interpretation. Although no formal sensitivity analysis was conducted, the overall pattern of findings did not appear to depend on the small subset of lower-scoring studies.

In terms of measurement, some studies used validated scales (e.g., Carral and Alcover, 2019; Marchiondo et al., 2019; Spoelma and Marchiondo, 2024), while others relied on ad hoc scales without addressing their psychometric properties (e.g., Zhang and Gibney, 2019), which can also undermine measurement quality. Regarding the study designs, an additional interpretive constraint should be noted. Only 6 of the 22 included studies used longitudinal designs, whereas cross-sectional studies dominated the remaining evidence base. The predominance of cross-sectional and correlational studies constitutes a limitation and requires caution when interpreting ageism as a causal determinant of workplace wellbeing. Because most studies collect data at a single point in time, temporal precedence cannot be established with confidence, and the observed associations may reflect competing explanations, including reverse causation and omitted-variable bias. For example, workers with poorer wellbeing may be more likely to perceive, recall, or report age-related mistreatment, just as exposure to ageism may contribute to diminished wellbeing.

It is also noteworthy that some studies also rely on age-group comparisons within cross-sectional data; chronological age and cohort membership are inherently confounded, making it difficult to determine whether observed differences reflect aging processes, historically specific generational experiences, or both. This ambiguity is particularly relevant in research on older workers, where age-related differences may also be shaped by cohort-specific work histories, institutional conditions, and normative expectations about later-life employment.

Various sampling-related challenges have been identified, including small, nonrepresentative sample sizes (e.g., Landrum, 2000) and recruitment biases (e.g., Choi et al., 2018). Some studies also inadequately stratify samples by age, often collapsing older participants into a single category (e.g., defining “older” simply as over 55 (Landrum, 2000) and thereby overlooking meaningful interindividual and intergroup variation. This limitation is not trivial because age is a continuous attribute and the boundaries of the “older worker” category are context-dependent (e.g., occupation, industry, and normative age expectations), underscoring the need for more fine-grained age groupings and clearer theoretical justification of age cut-offs.

As noted in the previous section, the evidence synthesized in this review comes primarily from North America, Europe, and parts of Asia. There is very limited representation from low- and middle-income or non-Anglophone settings. This geographic concentration introduces a clear location bias and limits the external validity of current findings. Cultural norms, labor-market structures, and institutional arrangements surrounding later-life work vary substantially across countries and welfare regimes (Pan American Health Organization [PAHO], 2021; Hansson et al., 2023; Jansen, 2017; Pienknagura and Evans, 2021). Although the overall direction of the association is likely to remain negative, its magnitude, salience, and underlying mechanisms are unlikely to be invariant across cultural and institutional contexts. The meaning of continued employment at older ages, the extent to which later-life work reflects choice rather than economic necessity, and the social value attached to older workers may shape how ageism is enacted, perceived, and translated into consequences for wellbeing.

This concern is reinforced by the fact that many of the conceptual frameworks and measurement tools used in the literature were developed in Western contexts. These may not capture ageism equivalently across cultures, thereby limiting the comparability and transferability of existing findings (Marrero-Vega et al., 2025). This matches broader criticism in the literature regarding the limited cross-cultural validity of ageism measures (Ayalon, 2018; Kang and Kim, 2022). As a result, the accuracy of estimates and the reliability of currently available measures may be compromised. The marked underrepresentation of regions such as Latin America leaves key contextual conditions insufficiently examined. This restricts what can be claimed about the generalizability and likely heterogeneity of the observed associations.

### 5.2 Limitations of the review processes

This review process also has limitations that should be acknowledged. First, the final sample was drawn almost entirely from peer-reviewed publications, with only one study identified as gray literature, and eligibility was limited to articles published in English or Spanish. This may have decreased the visibility of relevant evidence published in other languages or disseminated through less accessible outlets, thereby narrowing the range of perspectives included in the review and increasing the risk of publication bias. In particular, studies reporting null, weaker, or more ambiguous findings may have been less likely to be published and, therefore, less likely to be included in this review.

Second, although the search strategy was broad and systematic, implemented across multiple major databases, it remains possible that some relevant studies were not retrieved, as is often the case in reviews addressing conceptually diffuse constructs across heterogeneous literatures. Third, the evidence base synthesized here was overwhelmingly quantitative, with only one qualitative study included. This matters because the review is therefore better suited to identifying recurrent patterns of association than to capturing the contextual, interpretive, and experiential complexity through which workplace ageism may be understood and enacted in everyday organizational life.

Additionally, the lack of prospective protocol registration is a limitation of this review, as registering protocols improves methodological transparency and decreases the risk of bias in how the review is conducted and reported. Lastly, while the studies encompass various national contexts, there is a significant lack of research from Latin America, underscoring a notable geographical and cultural gap.

### 5.3 Implications and recommendations for future research

Despite these limitations, this review offers two interrelated theoretical contributions. First, it brings together a clear definition and an organizing framework that helps make sense of how ageism relates to wellbeing at work among older workers. In doing so, it aligns with and provides empirical grounding for the Pan American Health Organization [PAHO] (2021) view of ageism as operating across intrapersonal, intersubjective, and institutional levels, while also showing that the current evidence remains concentrated on the first two levels, leaving organizational/institutional manifestations comparatively underexamined.

Second, by mapping the mediators and moderators examined in the literature, the framework advances beyond a static view of the relationship between ageism and workplace wellbeing. It adopts a process model where (1) intrapersonal cognitive–affective mechanisms (such as self-perceptions, appraisals, rumination, aging anxiety) and (2) work-structural pathways centered on job demands and resources (including workload, emotional exhaustion, support, financial strain) help explain how ageism leads to reduced wellbeing at work. Additionally, this translation is most often intensified or mitigated by relational factors such as social support and the quality of supervisory relationships.

The practical implications of this review point to the need for an explicitly multilevel response to workplace ageism, one that combines structural change with targeted psychological and relational interventions. A useful way to organize these interventions is the Individual, Group, Leader, Organization (IGLO) framework (Day and Nielsen, 2017), because the mechanisms and moderators identified in the evidence fit well onto these levels.

At the individual level, interventions can focus on the intrapersonal mechanism through which ageism acts (e.g., appraisals, rumination, negative self-perceptions of age, and aging anxiety), strengthening coping and reframing processes that reduce the likelihood that ageist experiences translate into reduced wellbeing (Spoelma and Marchiondo, 2024; Takeuhi and Katagiri, 2024; von Hippel et al., 2019).

At the group level, the nature of moderation findings suggests that improving day-to-day intergenerational relationships is not just something “nice to have” but a protective resource, workplaces can intentionally increase high-quality cross-age contact and collaboration (e.g., structured opportunities for shared projects, spaces and routines that facilitate informal interaction, and mentoring designed as mutual learning rather than role-stereotyped support) (Yeung et al., 2021).

At the leader level, the evidence is especially clear regarding the importance of supervisory relations (Harada et al., 2019; Roscigno et al., 2022). Leaders and supervisors should be trained and held accountable for inclusive, age-fair management practices because supervisor support and the quality of supervisory relationships reliably condition whether age discrimination translates into lower wellbeing at work.

Finally, at the organizational level, interventions should target the structural conditions that allow exposure to ageism and shape job demands/resources. This includes age-inclusive HR practices, equitable access to development opportunities, workload and work-design decisions, and clear reporting/enforcement mechanisms; when these are in place, ageism is less likely to become a recurring demand and more likely to be countered by resources embedded in the system (Griffin et al., 2016; Thorsen et al., 2016; Yeung et al., 2021).

Future research directions emerge from the implications, discussion, and limitations identified in this review. First, there is a need for longitudinal studies to clarify the long-term effects of workplace ageism and to establish causal pathways linking exposure to ageism at work to workplace wellbeing outcomes. Second, further exploration of mediators and moderators is essential to unravel the mechanisms through which ageism operates and to identify key leverage points for more effective, evidence-based interventions.

Third, research should expand its focus to include institutional and organizational determinants (e.g., policies, work design, hiring and promotion practices, and HR policies) to elucidate how organizational factors shape and perpetuate ageist processes. Fourth, more research should be conducted in regions such as Latin America to identify context-dependent variables that link ageism to workplace wellbeing. At least, future investigations should adopt an intersectional approach to examine how ageism interacts with other forms of inequality (e.g., gender and race), thereby providing a more nuanced understanding of vulnerability and resilience among diverse worker populations.

## 6 Conclusion

Ageism in the workplace is a pervasive and complex phenomenon with significant negative implications for the workplace wellbeing of older workers. This relationship is mediated and moderated by multiple interpersonal, relational, and job-related factors, which constitute potential leverage points for evidence-based anti-ageism interventions. In the context of an aging workforce, the impact of ageism extends beyond individual experiences to shape social dynamics, organizational functioning, and performance outcomes. However, the latter was not our primary focus; the literature highlights significant findings in this domain.

While we acknowledge that ageism has gained increasing prominence on the research agenda in recent years, greater empirical and theoretical efforts are still required to address its manifestations in contemporary labor markets. The present systematization contributes to this endeavor by consolidating and evaluating the available evidence, thereby providing a stronger foundation for future studies to build on and move beyond the existing knowledge base. In conclusion, effectively addressing ageism in the workplace requires a coordinated approach that integrates research, organizational practices, and public policy to promote age equity.

## Data Availability

The original contributions presented in the study are included in the article/Supplementary material, further inquiries can be directed to the corresponding author.
