# Supplementary material for: Ageism toward older workers and wellbeing at work: a systematic review
Source: Front Psychol. 2026 Jun 10;17:1824641. doi: 10.3389/fpsyg.2026.1824641 (PMC13291071; doi:10.3389/fpsyg.2026.1824641)
Supplement: Supplementary file 1 [file Table_1.docx]

| **Supplemental Table S1** | | | | | | | |
| --- | --- | --- | --- | --- | --- | --- | --- |
| *Overview of the Studies Included* | | | | | | | |
| **Author(s)** | **Title** | **Country** | **Year of Publication** | **Total Sample Size** | **Mean Age / Distribution (Years)** | **Range of Ages** | **Gender Distribution** |
| Landrum | *Major concerns of workers at three stages of life.* | United States | 2000 | n=146 | 18-34 (37%)  35-54 (55%)  55+ (8%) | 18–55+ | 59% Women  41% men |
| Takeuhi & Katagiri. | *Effects of workplace ageism on negative perception of aging and subjective well-being of older adults according to gender and employment status.* | Japan | 2024 | n=600 | 65.7  (SD = 4.24) | 60–74 | 66.6 % Women  (n=400)  33.3% Men  (n=200) |
| Suberry & Bodner. | *Psychological Well-Being and Self-Aging Attitudes Moderate the Association between Subjective Age and Age Discrimination in the Workplace.* | Israel | 2024 | n=568 | 66.21  (SD = 11.95) | 50–95 | 55.8% Women  44.2% Men |
| Alam & Shin. | *A moderated mediation model of employee experienced diversity management: openness to experience, perceived visible diversity discrimination and job satisfaction.* | United States | 2020 | n=351 | -25 (14%) 25-40 (63%) 41-60 (21%) +60 (2%) | 25–60+ | 53.7% Women  46.3% Men |
| Mohamed & Shaban. | *Age and expertise: The effects of ageism on professional recognition for senior nurses.* | Saudi Arabia | 2024 | n=20 | 57.3  (SD = 4.43) | 50–65 | 70% Women  (n=14)  30% Men  (n=6) |
| Roscigno et al. | *Workplace Age Discrimination and Social-psychological Well-being.* | United States | 2022 | n= 3.379 | 51.38  (SD = 7.42) | 40–70 | 51.11% Women  48.89%  Men |
| Carral & Alcover. | *Measuring Age Discrimination at Work: Spanish Adaptation and Preliminary Validation of the Nordic Age Discrimination Scale (NADS).* | Spain | 2019 | n=209 | 58.6  (SD = 2.87) | 55–67 | 74.2% Women  (n=55)  25.8 % Men  (n=54) |
| Choi et al. | *Understanding work enjoyment among older workers: The significance of flexible work options and age discrimination in the workplace.* | United States | 2018 | n=5.702 | 58.91  (SD = 0.14) | 50–89 | 48 % Women  52% Men |
| Griffin et al. | *The longitudinal effects of perceived age discrimination on the job satisfaction and work withdrawal of older employees.* | Australia | 2016 | Sample 1:  n=726 (T1)  n=443 (T2)  n= 261 (T3)  Sample 2  n= 1,552 (T1)  n =1,246 (T2)  n =1,136 (T3) | Sample 1: 56.79  (SD = 6.92)  Sample 2:  62.86  (SD = 2.44) | - | Sample 1:  42.5% Women  57.5% Man  Sample 2:  48.6% Women  51.4% Men |
| Harada et al. | *Perceived Age Discrimination and Job Satisfaction Among Older Employed Men in Japan.* | Japan | 2019 | n=514 | 59.32  (SD = 2.99) | 55-64 | Only men  (100%) |
| Jelenko | *The role of intergenerational differentiation in perception of employee engagement and job satisfaction among older and younger employees in Slovenia.* | Slovenia | 2020 | n=1505 | Sample that meets criteria:  55-60 (33.3%)  60+ (4.8%) | 18 - 60+ | 54.9% Women  45.1% Men |
| Lee et al. | *The association between perceived discriminations and well-being in Korean employed workers: The 4th Korean working conditions survey.* | Korea | 2017 | n=32,984  (n >50: 10,352) | Sample that meets criteria:  50-59 (20.1%) to 60≥ (11.3%)  [n=10.351 (33.4%)] | 20 to 60≥ | 48.3% Women  51.7% Men |
| Macdonald & Levy. | *Ageism in the Workplace: The Role of Psychosocial Factors in Predicting Job Satisfaction, Commitment, and Engagement.* | United States | 2016 | n= 800 | 38.94  (SD = 14.21) | 18-75 | 68.7% Women (n=550)  31.3% Man (n=250) |
| Manzi et al. | *Age-based stereotype threat and negative outcomes in the workplace: Exploring the role of identity integration.* | Italy | 2019 | n=2.348 | 58  (SD = 3.7) | - | 60.3 % women (n=1,417)  39.7% men  (n=931) |
| Shippee et al. | *Long-Term Effects of Age Discrimination on Mental Health: The Role of Perceived Financial Strain.* | United States | 2019 | n=3.296 | 37.069  (SD= 4.358, measure on 1967) | 30-44 (measure on 1967) | Only women  (100%) |
| Spoelma & Marchiondo. | *A shield against ageism: Self-affirmation mitigates the negative effects of workplace age discrimination on well-being and performance.* | United States | 2024 | Study 1 -T1,2,3- (n=154)  Study 2 (n=198) -T1,2-  Study 3 (n=286) -T1,2,3-  Study 4 -T1,2- (n=84) | Study 1:  55.2% of participants were from the “young” age group:  M = 25.73  (SD = 3.42)  44.8% were from the “older” age group  M = 58.85  (SD = 5.41). | 18–30 and 50+ | Sample that meets criteria:  68% women;  32% man |
| Thorsen et al. | *Psychosocial work environment and retirement age: a prospective study of 1876 senior employees.* | Denmark | 2016 | n=1876  (436 from the general sample, 1122 from the senior sample, and 318 from the company sample from 164 different companies) | Age 56–58  (n= 994, 53%)  Age 59–61  (n= 669, 36%) Age 62–64  (n= 21, 11%) | 56-64 | 50% Women (n=938);  50% Men (n=938) |
| Von Hippel et al. | *Stereotype threat among older employees: Relationship with job attitude and turnover intentions.* | Australia | 2013 | Study 1: Sample 1 (n=602)  Sample 2 (n=473)  Study 2:  (n =401)  Sample 1 (older workers):  n = 202  Sample 2 (younger workers):  n = 199 | Study 1  56.79  (SD = 6.92)  Study 2  62.86  (SD = 2.44) | 50–75 | Study 1  42.5% Women  57.5% Men  Study 2  48.6% Women  51.4% Men |
| Von Hippel et al. | *Age-based stereotype threat and work outcomes: Stress appraisals and rumination as mediators.* | Australia | 2019 | n=280 | Overall:  39.35  (SD= 12.86) | 18-66 | Sample that meets criteria:  70.2% Women (n=59)  29.8%% Men (n=25) |
| Yeung et al. | *Perceived age discrimination in the workplace: the mediating roles of job resources and demands.* | China | 2021 | n=333 | 46.62  (SD=6.21) | 40-68 | 60.1% Women  39.9% Man |
| Zhang & Gibney. | *Ageism and perceived job sustainability: a comparative European analysis.* | 28 European Member states | 2019 | n=22.229 | 50.9 | 40–70 | 49% Women  51% Men |
| Marchiondo et al. | *Trajectories of perceived workplace age discrimination and long-term associations with mental, self-rated, and occupational health.* | United States | 2019 | n=3957 | 57.7  (SD = 6.4) | 60-87 | 56% Women  44% Men |
