# Supplementary material for: Ageism toward older workers and wellbeing at work: a systematic review
Source: Front Psychol. 2026 Jun 10;17:1824641. doi: 10.3389/fpsyg.2026.1824641 (PMC13291071; doi:10.3389/fpsyg.2026.1824641)
Supplement: Supplementary file 2 [file Table_2.docx]

| **Supplemental Table S2** | | |
| --- | --- | --- |
| *Main findings of the study on the relationship between ageism and workplace wellbeing.* | | |
| **Reference** | **Study Aim** | **Main findings in the relationship between ageism and workplace wellbeing.** |
| (Landrum, 2000) | Examines how perceived ageism, social support, sexism, racism, sexual harassment, job satisfaction, and mental health impact younger, middle-aged, and older workers. | Workers who perceived being treated differently because of their age reported higher anxiety and depression; perceived ageism correlated with state anxiety (r = .21), trait anxiety (r = .17), and depression (r = .25). In a stepwise regression predicting job satisfaction, the social support item was the strongest predictor, followed by job security and ageism. |
| (Takeuhi & Katagiri, 2024) | This study examined the effects of workplace ageism on attitudes toward aging and subjective well-being according to gender and employment status. | Seventy-five percent of participants perceived some form of workplace ageism. Among men currently working, workplace ageism showed direct associations with lower satisfaction with life (SWL) (β = −0.176, p < .05) and higher negative affect (NA) (β = 0.179, p < .05), plus indirect associations via negative self-perceptions of aging (SWL indirect β = −0.070, p < .01; NA indirect β = 1.000, p < .05). Among men not working, direct effects on SWLS/NA were non-significant (β = 0.004; β = 0.187), with a marginal indirect association with lower SWLS (β = −0.083, p < .10). For women whose longest employment was full-time, workplace ageism was linked to poorer well-being mainly indirectly: among those currently working, indirect effects were significant for SWL (β = −0.058, p < .05) and NA (β = 0.145, p < .05) despite non-significant direct paths; among those not working, an indirect association with higher NA remained (β = 0.094, p < .05). For women whose longest employment was part-time, there were no significant direct or indirect effects among those currently working, whereas among those not working ageism related to higher NA both directly (β = 0.256, p < .05) and indirectly (β = 0.133, p < .05), with a marginal indirect association with lower SWL (β = −0.056, p < .10). |
| (Suberry & Bodner, 2024) | Explores the relationships between subjective age, self-aging attitudes, psychological well-being, and age discrimination in the workplace and the moderating effects of self-aging attitudes and psychological well-being on the link between subjective age and age-related discrimination. | Psychological well-being was consistently associated with lower perceived workplace age discrimination. Specifically, well-being showed a moderate-to-strong negative correlation with perceived age discrimination (r = −0.45, p < .001) and remained a significant negative predictor in hierarchical regression after controlling for demographic covariates (β = −0.307, p < .001). |
| (Alam & Shin, 2020) | Analyzes how workplace diversity management affects job satisfaction through perceived discrimination, focusing on employees’ openness to experience. | Perceived age discrimination was negatively correlated with job satisfaction (*r* = –0.31, *p* < .01). |
| (Mohamed & Shaban, 2024) | Examines the perceptions of older nurses regarding age discrimination and recognition in their profession, with a focus on the impact of age-related biases on career advancement, job satisfaction, and self-perception. | Age-related biases lead to significant professional marginalization, reduced job satisfaction, and emotional distress among senior nurses (qualitative study). |
| (Roscigno et al., 2022) | Examines the prevalence and effects of workplace age discrimination in full-time workers aged 40 and older. | Over 6.5% of full-time workers aged 40–70 reported age discrimination, with prevalence rising from 4–11% (ages 40–50) to 22% (age 60) and 36% (age 70). Perceived age discrimination was positively associated with job-related stress (β = 0.313, *p*<.001) and poor mental health (β = = 2.497, *p*<.001). |
| (von Hippel et al., 2013) | Investigates the impact of stereotype threat on older employees by examining how the belief in being subjected to negative stereotypes influences their job attitudes (specifically job satisfaction and organizational commitment), work mental health, and intentions to resign or retire (Study 1). Furthermore, the study seeks to determine whether these relationships are distinct for older employees compared to younger employees (Study 2). | In study 1 (older employees; two samples) stereotype threat was strongly negatively correlated with job satisfaction (r = –0.40 to –0.37) and work-related mental health (r = –0.43 to –0.36). In study 2 (older vs. younger employees), among older adults, correlations were stronger (r = –0.40 for job satisfaction; r = –0.50 for mental health, p < .001), while younger adults showed weaker associations (r = –0.15 and r = –0.16, p < .05). |
| (Carral & Alcover, 2019) | Adapts and validates the Nordic Age Discrimination Scale (NADS) for older workers and explores its relationship with age, discrimination, bullying, health, well-being, work ability, social support, and retirement intentions. | Experiences of age discrimination were negatively correlated with job and life satisfaction (r=–0.20 con p<0.01). |
| (Choi et al., 2018) | Explores workplace determinants (human resources management-related factors) of work enjoyment among older workers. | The odds of rating enjoyment in a higher category were 33% lower for each unit increase in promotion preference for younger workers (OR = 0.67, p < .0001). |
| (Griffin et al., 2016) | Investigates the long-term impacts of perceived age discrimination on older workers' job attitudes and behaviors, specifically its effects on job satisfaction, withdrawal, and retirement decisions. | The study used two 3-wave panel designs of older employees with short (3-week) and long (1-year) lags to examine perceived age discrimination and job satisfaction (as well as withdrawal/retirement outcomes). In both samples, perceived age discrimination was consistently negatively associated with job satisfaction at each wave (Sample 1: r = −.35, −.44, −.43; Sample 2: r = −.21, −.23, −.19; all p < .01), with stronger associations in the short-lag sample. Cross-wave correlations were also negative (Sample 1: r = −.39, −.38; Sample 2: r = −.18, −.20). Cross-lagged analyses indicated that effects were primarily evident from Time 1 to Time 2, whereas Time 2–Time 3 paths were only marginal (p < .075). A change (trajectory) analysis further showed that increases in perceived age discrimination predicted lower Time 3 job satisfaction in the short-lag sample (β = −.08, p < .05), but not in the long-lag sample. |
| (Harada et al., 2019) | Investigates how age discrimination affects job satisfaction in older employed men in Japan and the role of psychosocial resources in mitigating this impact. | Perceived age discrimination was significantly negatively correlated with job satisfaction (r = –0.27, p < .001) and also showed a significant negative effect in regression analysis (β = –0.18, p < .001). |
| (Jelenko 2020) | Examines the relationship between intergenerational differentiation, specifically age discrimination, and its effects on job satisfaction and employee engagement. | Intergenerational differentiation (age discrimination) had a direct negative effect on job satisfaction, with a stronger impact among older employees (β = –0.41) than younger ones (β = –0.32). |
| (Lee et al., 2017) | Examines the association between perceived discrimination and well-being. | Exposure to age discrimination was linked to a 41% increased rate of poor well-being (IRR: 1.41, 95% CI: 1.28–1.56) and an Odds Ratio (OR) of 1.51 (95% CI: 1.36–1.68). More females (6.6%) reported experiencing age discrimination than males (5.4%). In the study, the number of incidents (0 to 3) refers to the count of different types of discrimination experienced. The risk of poor well-being rose with the number of incidents: OR´s were 1.32 for one incident, 1.69 for two, and 2.60 for three. |
| (Macdonald & Levy, 2016) | Examines how psychosocial factors, such as age identity, aging anxiety, perceived age discrimination, social support at work, and work centrality; impact job satisfaction, commitment, and engagement. It also aims to identify buffers (e.g., age identity, social support) and hindrances (e.g., age discrimination, aging anxiety) affecting job longevity. | Perceived age discrimination was negatively associated with job satisfaction (r = –0.18; β = –0.13), though it did not significantly predict job commitment or engagement in regression models. Aging anxiety unlike perceived discrimination (which only predicted satisfaction), significantly predicted reductions in all three dimensions: job satisfaction (β= -0.15, p < .001), organizational commitment (β = -0.11, p < .05), and engagement (β= -0.11, p < .05). |
| (Manzi et al., 2019) | Explores mediators and moderators of the relationship between age-based stereotype threat and work and individual adjustment. | Perceived age-based stereotype threat was higher for women (M = 2.46, SD = 0.89) than for men (M = 2.31, SD = 0.83), as indicated by a significant gender effect, F(1, 2,342) = 9.388, p = .002. No significant differences were observed across job status, nor was the gender × job status interaction significant. Threat negatively related to flourishing (β = -0.13 [-.18, -.09] p < .01). An indirect effect on flourishing was proven via work–age identity, with a coefficient of β = -0.08 (95% CI [-0.09, -0.06]) |
| (Shippee et al., 2019) | Examines the role of work-related perceived age discrimination on women’s mental health over the life course and tests whether financial strain mediates this relationship. | Women who experienced work-related age discrimination reported higher depressive symptoms and lower life satisfaction over the life course (β = –0.084, p < .05). Perceived financial strain was a strong and significant predictor of depressive symptoms and fully mediated the association between age discrimination and depressive outcomes (z = 3.64, p < .001), rendering the direct effect of age discrimination on depressive symptoms non-significant when financial strain was included in the model. In contrast, women exposed to age discrimination had significantly lower odds of reporting high life satisfaction (OR = 0.539, p < .01), with financial strain partially mediating this relationship, while age discrimination remained a significant predictor of life satisfaction. |
| (Spoelma & Marchiondo, 2024) | Explores how a brief personal values affirmation intervention can reduce the adverse impacts of age discrimination in the workplace on employee well-being and performance. | Perceived workplace age discrimination was positively associated with threat appraisal (r = 0.29, p < .01), somatic complaints (r = 0.25, p < .01), and emotional exhaustion (r = 0.25, p < .01). In regression models, it significantly predicted threat appraisal (β = 0.31, p < .01) and somatic complaints (β = 0.25, p < .01), but not emotional exhaustion (β = 0.17). A personal values affirmation intervention moderated the relationship between age discrimination and threat appraisal, with significant effects observed in the control condition but not in the intervention condition. |
| (Thorsen et al., 2016) | Identifies psychosocial work environment factors important for retaining senior employees by examining their association with voluntary early retirement in a longitudinal study. | Job satisfaction was negatively correlated with perceived age discrimination (r = –0.24, p < .0001). Age discrimination was a significant predictor of early retirement, with a hazard ratio (HR) of 1.58 (p < 0.01), even after controlling for job satisfaction. Among the factors considered, low job satisfaction emerged as the strongest predictor of early retirement, exhibiting an HR of 3.33 (p < 0.0001). |
| (von Hippel et al., 2019) | Explores stress appraisals and rumination as mechanisms through which age-based stereotype threat negatively affects older employees. | Within-person correlations showed no significant association between stereotype threat events and job satisfaction (r = 0.00) or workplace well-being (r = –0.05). In regression models, stereotype threat events did not significantly predict job satisfaction (β = –0.03, p = 0.709), but were associated with a significant decrease in workplace well-being (β = –0.14, p = 0.018). Age significantly moderated the relationship between stereotype threat events and both job satisfaction (β = –0.01, p = 0.032) and workplace well-being (β = –0.02, p < .001). Simple slopes analysis revealed no significant effect of stereotype threat events on job satisfaction for younger (β = 0.15, p = 0.222) or older employees (β = –0.22, p = 0.090). However, stereotype threat events significantly reduced workplace well-being among older employees (β = –0.35, p < .001), with no significant effects observed for younger employees (β = 0.06, p = 0.424). |
| (Yeung et al., 2021) | Investigates the harmful effects of perceived age discrimination on work-related outcomes. | Correlational results showed that perceived age discrimination (PAD) was positively associated with work strain (r = 0.22, p < .001), while its associations with work engagement (r = −0.10, p = .068) and intention to stay (r = −0.09, p = .099) were negative but non-significant. PAD was also related to lower job resources (job autonomy: r = −0.11, p = .043; support: r = −0.10, p = .059) and higher job demands (emotional demand: r = 0.20, p < .001; workload: r = 0.10, p = .060), and PAD was not significantly related to chronological age (r = 0.07, p = .205) within this 40+ sample. |
| (Zhang & Gibney, 2019) | Explores the link between ageism in the workplace and job sustainability perceptions among European workers aged 40 and over, in the context of active aging strategies. | Workers reporting workplace age discrimination showed a higher prevalence of poor mental well-being (0.327 vs. 0.171; p < .001) and lower work satisfaction (means 58.36 vs. 67.85; p < .001). In generalized SEM models predicting perceived job sustainability, experienced age discrimination was negatively associated with sustainability in the baseline sample (β = −0.692, p < .01) and remained negative among older workers (β = −0.198 to −0.258, p < .10), indicating lower likelihood of perceiving one can sustain work to older ages. |
| (Marchiondo et al., 2019) | Examines older employees' experiences of perceived workplace age discrimination and its long-term effects on their mental health, self-rated health, job satisfaction, and likelihood of working beyond retirement age. | An initial measure of perceived workplace age discrimination was negatively correlated with job satisfaction, with a coefficient of -0.286 (p < 0.01). Furthermore, changes in perceived age discrimination were negatively related to changes in job satisfaction over time, with a coefficient of -0.046 (p < 0,05), indicating statistical significance. Increases in age discrimination were significantly related to increases in depressive symptoms (r = 0.948, p < .01). |
